# Multiple Mechanisms of Flaxseed: Effectiveness in Inflammatory Bowel Disease

**DOI:** 10.1155/2020/7974835

**Published:** 2020-07-12

**Authors:** Amber Hanif Palla, Anwar-ul-Hassan Gilani, Samra Bashir, Najeeb Ur Rehman

**Affiliations:** ^1^Faculty of Pharmacy, Department of Basic Medical Sciences, Barrett Hodgson University, Karachi, Pakistan; ^2^Department of Biological and Biomedical Sciences, The Aga Khan University Medical College, Karachi, Pakistan; ^3^The University of Haripur, Haripur, Khyber Pakhtunkhwa, Pakistan; ^4^Department of Pharmacy, Capital University of Science and Technology, Islamabad, Pakistan; ^5^Department of Pharmacology, College of Pharmacy, Prince Sattam Bin Abdulaziz University, Al-Kharj 11942, Saudi Arabia

## Abstract

**Materials and Methods:**

Aqueous-methanolic crude extracts of Flaxseed (Fs.Cr) and Flaxseed oil were tested against 6% acetic acid- (AA-) induced colitis in BALB/c mice. Microscopic damage parameters of the hematoxylin and eosin-stained and periodic acid-Schiff-alcian blue-stained sections of the colon were scored to be assessed. Possible antispasmodic mechanism was studied on isolated rabbit jejunum, while antibacterial activity was assessed *in vitro* for microbes implicated in IBD.

**Results:**

In AA-induced colitis, Flaxseed oil was found to be more effective in reducing mortality and colonic ulcers than Fs.Cr at 500 mg/kg dose. Fs.Cr was more efficacious in increasing mucin content as compared to oil, exhibiting slightly greater anti-inflammatory effect (50% vs 35%) and reducing depth of lesion (55% vs 42.31%, respectively). Antispasmodic activity of Fs.Cr (0.03 and 0.1 mg/ml) was mediated by phosphodiesterase inhibitors (PDEI, possibly PDE-4 subtype) with a resultant increase in cAMP levels. Flaxseed oil PDEI activity was mild (1 and 3 mg/ml). Fs.Cr (0.1 and 0.3 mg/ml) was potent in exhibiting anticholinergic activity, similar to dicyclomine, whereas Flaxseed oil showed anticholinergic effect at 1 and 3 mg/ml. Flaxseed oil (9 and 14 *µ*g/ml) was bactericidal against enteropathogenic *E.coli* (EPEC), enterotoxigenic *E.coli* (ETEC), and enteroaggregative *E.coli* (EAEC), whereas Fs.Cr exhibited bactericidal effect against EPEC at 100 *µ*g/ml.

**Conclusions:**

Results of this study, taken together with previous studies, suggest that Flaxseed possesses anti-inflammatory, antibacterial, and antispasmodic action through multiple pathways and thus offers promising potential to be developed for IBD.

## 1. Introduction

Inflammatory bowel disease (IBD) is a heterogeneous disease in which multiple triggers act simultaneously. The major maneuvering targets in IBD are immune dysregulation, barrier, and microbial defects, along with abdominal spasms and colic as accompanying symptoms [1]. Despite advancement in IBD therapeutics, rate of failure to attain the remission remains high possibly due to multifactorial etiology of the disease.

Flaxseed (scientific name: *Linum usitatissimum*) has traditionally been used for IBD [2, 3]. In our earlier studies, we provided some evidence for its traditional use by showing that the aqueous-methanolic crude extract of Flaxseed (Fs.Cr) ameliorated the severity in mice model of colitis by reducing mediators of inflammation (myeloperoxidase and cytokines) and inducers of oxidative stress [4]. The extract that we studied comprised predominantly of polar components, whereas Flaxseed as a whole is a combination of both the polar and nonpolar constituents [5]. Therefore, to gain an insight into the holistic potential of Flaxseed in IBD, one of the aims of the current study was to assess the effect of Flaxseed oil (nonpolar constituents) for its effectiveness against IBD model of mice and, subsequently, compare these parameters with Fs.Cr.

Knowing that antispasmodics have an important role in IBD therapeutics as an adjunctive therapy [1], we also aimed to explore the precise mode of antispasmodic mechanisms of Flaxseed (both the extract and oil) using isolated gut tissues from experimental animals. Earlier, we have reported that both Fs.Cr and Flaxseed oil exhibited antidiarrheal activity with possible involvement of Ca^+2^ channel inhibition and nonspecific K^+^ channel activation, respectively [6, 7]. However, the possibility of other potential mechanisms was there as the inhibitory effect in isolated jejunal tissues against carbachol- (CCh, 1 *μ*M) induced contractions was not studied, which can help in identifying the presence of anticholinergic and/or phosphodiesterase inhibitory mechanisms [8, 9], both of which have potential for IBD therapeutics [10, 11].

The third aim of our study was to assess the antibacterial activity of Flaxseed against the bacteria implicated in IBD as microbes have an important role in IBD pathogenesis [12]. Amongst them, enteropathogenic *E. coli* (EPEC), enterotoxigenic *E.coli* (ETEC), and enteroaggregative *E.coli* (EAEC) are implicated in IBD as they share virulence properties with adherent invasive *E.coli* (AIEC) [13], a strain isolated from biopsies of ileal Crohn's disease (CD) patients. Flaxseed has not been studied for its activity against these pathogens. Hence, in the current study, efficacy and potency of Fs.Cr and Flaxseed oil against EPEC, ETEC, and EAEC were tested *in vitro*.

To sum up, our objective in the current study is to assess the holistic potential of Flaxseed by examining and comparing both the extract and the oil for their protective effect against acetic acid (AA)-induced colitis in mice, antispasmodic mechanisms, and antibacterial activity, thus encompassing the different triggers of IBD.

## 2. Materials and Methods

### 2.1. Standard Drugs

Acetic acid, acetylcholine chloride, carbamoyl choline chloride, DPX, periodic acid, prednisolone, Schiff reagent, and verapamil hydrochloride were purchased from Sigma Chemicals Co, St. Louis, MO, USA. Nutrient agar and Luria-Bertani broth powders used for antibacterial assays were also purchased from Sigma Chemicals. Chemicals used for making physiological salt solutions, including potassium chloride, calcium chloride, glucose, magnesium chloride, magnesium sulfate, potassium chloride, sodium bicarbonate, sodium dihydrogen phosphate, EGTA, and sodium chloride, were obtained from Merck, Darmstadt, Germany. DMSO and Tween-80 used for solubilization were purchased from Merck, Darmstadt, Germany, and Scharlau Chemicals, Barcelona, Spain, respectively. All chemicals used were of the available analytical grade. Cenogenics single strip kit was used for the occult blood test. cAMP enzyme immunoassay kit for multiple species was purchased from Arbor Assays, Ann Arbor, MI, USA. The reference drugs gentamycin was purchased from Sigma Chemicals Co, St. Louis, MO, USA. Nutrient agar and Luria-Bertani broth powders used for antimicrobial assays were purchased from Sigma Chemicals and were of the available analytical grade.

### 2.2. Preparation of Plant Material (Flaxseed Extract and Oil)

*Linum usitatissimum.* L (Flaxseed) was bought from an authentic herb supplier in the local market of Karachi, Pakistan. The plant name and physical characteristics matched the description when checked with http://www.theplantlist.org. It appeared as deep brown flat and oval seed with a pointed tip and measured about 4 mm. A sample of Flaxseed was deposited in the herbarium of the Department of Biological and Biomedical Sciences of the Aga Khan University, with a voucher number LU-SE-0812-106. The seeds were made free of dirt and other adulterants and were grounded finely into powder by an electrical chopper.

The aqueous-methanolic crude extract of Flaxseed (Fs.Cr) was prepared from approximately 1 kg ground Flaxseed by the process of cold maceration using aqueous methanol (30 : 70 v/v) as a solvent for extraction. [7]. For oil extraction, previously defined methods were used [14]. Briefly, approximately 1 kg of ground Flaxseed was soaked in petroleum ether and kept on a water bath at 45°C for 7 days with occasional shaking. Petroleum ether was evaporated using Rota vapor, and the oil was filtered to clarity. Respective yields of the Fs.Cr and the oil were 8 and 27% w/w with reference to dried seeds. Both the extract and the oil were plated on nutrient agar and incubated at 37°C for 24 hrs. There was no evident growth on the plate.

### 2.3. Animals

The study protocol (005-Ani-BBS-13) was approved by ECACU (Ethics Committee for Animal Care and Use) of the Aga Khan University, which is in accordance with the international guidelines for laboratory animal use and care [15]. Rabbits (1–1.5 kg) of either sex and of the local breed were used for the *ex vivo* study, whereas Sprague Dawley rats (150–200 g) were used for the *in vivo* study. Female BALB/c mice, 6–8 weeks old (22–30 g), were used for the colitis study and kept in the animal house of the Aga Khan University (Karachi), in rectangular cages (47 × 34 × 18 cm^3^) with sawdust (changed every 48 hrs). All animals were given a standard diet and maintained under standard conditions (temperature 22–25°C, 70–75% humidity, and 12 hr light and dark cycle). Animals also had free access to water and food ad libitum, throughout the study, except in case of induction of colitis, where animals' food was withdrawn before 18 hrs and rabbits were kept on fasting for 24 hrs before sacrificing them for *ex vivo* study.

Mice used for colitis study were sacrificed by cervical dislocation 24 hrs after induction of colitis, whereas rabbits starved for 24 hrs were anaesthetized with thiopental sodium (200 mg/kg, i.p.) and sacrificed by cervical dislocation. Bacterial strains used in this study included enteropathogenic *E. coli* (2348/69, EPEC), enterotoxigenic *E. coli* (H10407, ETEC), and enteroaggregative *E. coli*. (042, EAEC), grown aerobically at 37°C in Luria-Bertani broth (LB) for 16 hrs.

### 2.4. Working Stocks

For the *in vivo* and *ex vivo* experiments, the extract and the oil were solubilized in 10% DMSO-5% Tween-80, and subsequent dilutions were made in distilled water on the day of the experiment.

For determining the effect against enteric pathogens, the Fs.Cr stock was prepared as 1000 mg/ml in 50% methanol and then centrifuged at 14,000 g for 60 min. The supernatant was microfiltered, and subsequently a working stock was prepared into 1 mg/ml. From this working stock, Fs.Cr 10 and 20 *μ*l were picked for the antimicrobial assay (assay volume = 200 *μ*l) to get the total concentration to be 50 *μ*g/ml and 100 *μ*g/ml, respectively.

For positive control groups (100% kill), 10 *μ*l of microbes was suspended with 100 *μ*g/ml of vancomycin (for Gram-negative), whereas, for negative control, 10 *μ*l of inoculum was suspended with the corresponding volume of the vehicle solvent (MeOH). 12.5% methanol, corresponding to the final concentration of MeOH in Fs.Cr working solutions, was used as vehicle control. At this concentration, there was no effect on bacteria kill.

Flaxseed Oil was already in the liquid form and could be easily drawn in minutest quantities; hence, no dilution was required. 1 ml (1000 *μ*l) of oil corresponding to 1 mg (1000 *μ*g) was centrifuged at 14000 g for 60 min and subsequently microfiltered. From this, 1.8 *μ*l and 2.8 *μ*l were picked for assay (assay volume = 200 *μ*l) that corresponded to 9 *μ*g/ml and 14 *μ*g/ml, respectively (1.8 *μ*g in 200 *μ*l; for 1000 *μ*l or 1 ml, 1.8/200^∗^ 1000 = 9 *μ*g/ml).

### 2.5. Phytochemical Analysis

The aqueous-methanolic crude (30 : 70) extract of Flaxseed (Fs.Cr) was qualitatively analyzed according to standard methods for the presence or absence of alkaloids, anthraquinones, coumarins, flavonoids, saponins, sterols, tannins, and terpenes [16].

### 2.6. Experimental Protocol

The study design was similar to our previously reported study [4] with a sham control group, diseased control (untreated), positive control (prednisolone), and treatment groups. There were 12 animals in each group. Three treatment groups were administered 150, 300, and 500 mg/kg Fs.Cr, and another three groups were given similar doses of Flaxseed oil intraperitoneally (i.p.) for 5 days prior to the induction of colitis. On the 6^th^ day, all groups except the sham control group were induced with colitis under anaesthesia. All the groups continued to receive the respective treatments till the last day when mice were to be sacrificed.

Acetic acid- (AA-) induced colitis model by intrarectal (IR) administration was adopted to decipher the effect of Fs.Cr and oil, as reported previously [4, 17]. Briefly, BALB/c mice fasted for 18 hrs with access to water ad libitum were anaesthetized and administered 0.1 ml of 6% IR acetic acid (v/v, in 0.9% saline) through feeding needle, held vertically for 45 sec and then flushed with 0.1 ml of saline. Mice were sacrificed 24 hrs after the induction of colitis.

### 2.7. Evaluation of AA-Induced Colitis

#### 2.7.1. Mortality Rate and DAI

The numbers of mice dying in the respective groups were compared to the total animals present in the same group before start of the experiment, and mortality rate was calculated as follows:

(1) $$\begin{array}{c} \text{mortality Rate }=\frac{\text{mice died in a group}}{\text{total number of mice in that group}}\text{x }100. \end{array}$$

Disease activity index (DAI) was assessed after 24 hrs, which was the average of loss in body weight, effect on stool consistency, and type of rectal bleed [18]. The semiquantitative scoring details are elaborated in Table 1.

#### 2.7.2. Histological Assessment

After assessing DAI, mice were sacrificed by cervical dislocation, and the colon tissues, 5 cm proximal to anus, were excised and stored in neutral buffered formalin for 24 hrs. Histological processing of colon tissues was done to assess colonic damage using hematoxylin and eosin (H and E) staining, and mucus content was assessed by periodic acid-Schiff-alcian blue (PAS-AB) staining. For quantifying the microscopic damage of H and E and PAS-AB, stained sections of the colon were scored in a single blinded fashion by a histopathologist using Neurath et al. [19] and Vilaseca score [20], whereas goblet cells were assessed on the basis of level of the mucin staining according to Dorofeyev et al. [21], the details of which are presented in Supplementary Table 1.

### 2.8. Antispasmodic Mechanisms

Fs.Cr and Flaxseed oil were tested for their antispasmodic mechanisms using isolated rabbit jejunum preparations in a tissue bath assembly set-up, for their inhibitory effect against CCh- (1 *μ*M) induced contractions, and for their previously reported effect on spontaneous and high K^+^- [7] and low K^+^-induced contractions [6].

#### 2.8.1. PDE Inhibitory Activity

To assess PDE inhibitory effect of test materials, isoprenaline-induced inhibitory CRCs were constructed against CCh- (1 *µ*M) induced sustained contractions in isolated rabbit jejunum in the absence and presence of the Fs.Cr and Flaxseed oil, as described previously [22]. Since the PDEI activity of Fs.Cr was stronger as compared to oil, Fs.Cr was further studied for its inhibitory effect against the subtype of PDE enzyme along with cAMP levels in Fs.Cr tested tissues. For this purpose, a relationship between inhibitory effects of different PDE enzyme inhibitors was examined in the presence and absence of Fs.Cr [23, 24]. Briefly, isolated rabbit jejunal tissues were contracted by CCh (1 *µ*M). Once these contractions were sustained, concentration-dependent inhibitory responses were constructed by cumulative dosing of PDE enzyme subtypes 3, 4, and 5 inhibitors: cilostazol, rolipram, and zaprinast, respectively [25]. This was followed by restabilization of the jejunal tissues, and then the same types of concentration-dependent inhibitory responses of each of the PDE subtypes 3, 4, and 5 inhibitors were constructed in the presence of 0.03 mg/ml of Fs.Cr, or one of the other types of PDE inhibitors: cilostazol (100 *μ*M), rolipram (3 *μ*M), and zaprinast (100 *μ*M) (administered before inducing CCh-induced contractions). The results were interpreted based on the potentiation of PDE inhibitor's concentration-dependent inhibitory responses with or without pretreatment of Fs.Cr.

To confirm the PDE inhibitory effect of Fs.Cr, cAMP content of the jejunum was measured by enzyme immunoassay [26] using cAMP enzyme immunoassay kit for multiple species (Arbor Assays DetectX, Direct cAMP Enzyme Immunoassay kit, Ann Arbor, MI, USA). For this purpose, CCh-induced contraction of the jejunum was inhibited with Fs.Cr in respective different doses. Then the jejunal tissues were snap-frozen in liquid nitrogen; subsequently homogenized with 1 ml of sample diluent for every 100 mg of tissue, on ice; kept like this for 10 min; and then centrifuged at ≥6000 g at 4°C for 15 min. The supernatant was collected and either analyzed immediately or stored frozen at −80°C. Similar steps were repeated for tissues treated with different doses of papaverine, which served as a positive control, and tissues without administration of any drug and only contracted with CCh, which served as a negative control. The cAMP content was expressed as picomole per ml.

#### 2.8.2. Anticholinergic Activity

In order to confirm the involvement of anticholinergic mechanisms, first control concentration-response curves (CRCs) of Ach were constructed in guinea pig ileum up to the maximal effect, and then these CRCs were reconstructed in the presence of an increasing concentration of the plant material (Fs.Cr and oil). The rightward parallel shift in the CRCs of Ach indicated the competitive antagonism [27], while nonparallel displacement with suppression of the maximal response confirmed the nonspecific antagonism. [28].

### 2.9. Antibacterial Assay

EPEC, ETEC, and EAEC were assessed using the quantitative antibacterial assays as previously reported [29]. At first, optical density (OD) of bacteria was adjusted through Luria Broth (LB) to 0.22 corresponding to approximately 10^6^ colony forming units (c.f.u.), followed by making a serial dilution of bacteria at 10^−1^–10^−6^ and plating them; this became the inoculum control. Simultaneously, in other sets of tubes, 10 *µ*l of respective bacterial inoculum was suspended with different doses of Flaxseed oil (9 *μ*g/ml and 14 *μ*g/ml) and Fs.Cr (50 and 100 *µ*g/ml); the total assay volume was 200 *µ*l, and LB was used as an assay buffer. For negative control, 10 *µ*l of original inoculum (OI) was suspended with corresponding volumes of LB instead of Flaxseed oil/Fs.Cr. Additional negative control of the corresponding concentration of methanol (MeOH) was used for Fs.Cr, as it was solubilized in MeOH. For positive control groups (100% kill), 10 *µ*l of inoculum was suspended with 100 *µ*g/ml gentamycin. The assay tubes were then incubated in CO_2_ incubator for 2 hrs on shaking. After incubation, microbial cultures were 10-fold serially diluted in LB (10^−1^–10^−6^), plated on nutrient agar plates, and incubated further at 37°C overnight, and microbial c.f.u. were enumerated.

The effect of test material was compared to the originally incubated number of bacteria in terms of reduction in c.f.u. in respective treatment groups. The effect was considered as bactericidal if the concentration of bacteria in the treated groups was less than OI. The percent bactericidal effects were determined as follows:

(2) $$\begin{array}{c} 100-\left[\left(\frac{\text{c}.\text{f}.\text{u}.\text{ in extract}}{\text{original inoculum}}\right)*100\right]. \end{array}$$

The conclusions were drawn based on colony count as follows:

1. If the microbial c.f.u. was less than the c.f.u. in OI, it was considered as a bactericidal effect
2. If the microbial c.f.u. was similar to OI or greater than OI, it was inferred that test material has no effect

### 2.10. Statistical Analysis

The data expressed are means ± standard error of the mean (SEM; *n*: number of experiments). One-way ANOVA was applied to differentiate the results in cyclic AMP (cAMP) assay. A probability of *p* < 0.05 was considered significant. The concentration-response curves (CRCs) were analyzed by nonlinear regression using the GraphPad program (GraphPad, San Diego, CA, USA). The antibacterial analysis was determined by unpaired *t*-test (two-tailed) or one-way ANOVA followed by Tukey's posttest. All other graphing, calculations, and statistical analyses were also carried out using GraphPad software (GraphPad, San Diego, California, USA).

## 3. Results

### 3.1. Phytochemistry

Preliminary phytochemical analysis of the aqueous-methanolic crude extract of Flaxseed (Fs.Cr) revealed the presence of tannins, flavonoids, triterpenes, alkaloids, and coumarins, whereas saponins were weak and anthraquinones were not detected.

### 3.2. Evaluation of the Effect on the Ulcerative Colitis Model

The effects of Flaxseed oil and Fs.Cr at the doses of 150, 300, and 500 mg/kg were tested against AA-induced colitis.

#### 3.2.1. Mortality Rate and DAI

The mortality rate of 6% AA-induced colitis mice (untreated) was 58%, whereas pretreatment with Flaxseed oil and extract reduced the mortality rate significantly (*p* < 0.01 vs *p* < 0.001 at 300 and 500 mg/kg doses, respectively). The optimal effect of Fs.Cr was at 300 mg/kg dose, whereas Flaxseed oil's optimal effect was evident at 500 mg/kg dose. The effect of oil at 500 mg/kg dose was superior to that of the extract (8.33 ± 5% vs 33 ± 6%).

Overall, both the extract and oil improved the DAI significantly as compared to diseased control. Fs.Cr improvement in DAI started at 150 mg/kg, whereas Flaxseed oil improved DAI at 300 mg/kg dose. Both Flaxseed oil and extract were effective at 300 mg/kg doses, with Fs.Cr being more effective at this dose, whereas Flaxseed oil exhibited maximum improvement in DAI at 500 mg/kg dose (Table 2).

#### 3.2.2. Microscopic Damage

Both the extract and oil were equally efficacious in improving the microscopic damage parameters when compared to diseased control. However, the type of protective effect was different for both Fs.Cr and Flaxseed oil.

Both Flaxseed oil and Fs.Cr were equally efficacious in reducing neutrophil infiltration (Neurath score) except that Fs.Cr was more potent than Flaxseed oil. The colonic damage scores showed significant dose-dependent reduction by Fs.Cr, with maximum reduction by 55.18% at 300 mg/kg dose, and the oil also showed similar efficacy (55.89% reduction in Neurath score) but at 500 mg/kg dose. When the microscopic damage was quantified using Vilaseca score [20], which is the sum of the size of ulceration, inflammation, and depth of lesion, Flaxseed oil was more efficacious in reducing ulcers (max effect: 77.5% at 500 mg/kg dose) as compared to Fs.Cr (max effect: 56% at 300 mg/kg dose). Fs.Cr was slightly more efficacious than Flaxseed oil in reducing the inflammation (50% vs 35.46% respectively) and depth of lesion (55% vs 42.31% respectively).

The effects of Flaxseed extract and oil were compared for their effectiveness in increasing the mucin score, as compared to diseased control. Fs.Cr was both potent and efficacious (92% mucin increase) in promoting mucin production as compared to oil (maximum mucin production: 42.28%). The percentage difference is elaborated in Table 3, whereas the scores are illustrated in Supplementary Table 2.

### 3.3. Antispasmodic Mechanisms

Both Fs.Cr and Flaxseed oil were studied for their effect against CCh-, high K^+^-, and low K^+^-induced contractions in a spontaneously contracting rabbit jejunum, to assess and confirm their possible antispasmodic mechanisms.

**Figure 1:**
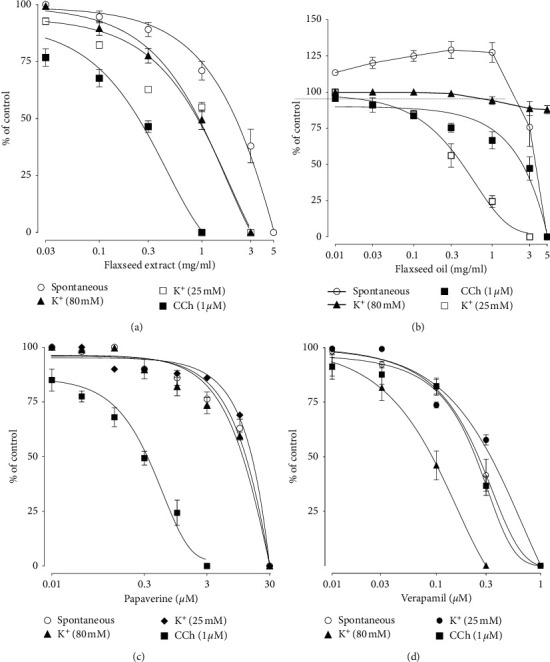
Inhibitory effect of aqueous-methanolic crude extract of Flaxseed (Fs.Cr) (a), Flaxseed oil (b), papaverine (c), and verapamil (d) against CCh-, high K^+^- (80 mM), and low K^+^- (25 mM) induced contractions in isolated rabbit jejunal preparations.

When compared for inhibitory effect against different spasmogens, Fs.Cr showed selectivity for CCh-induced contractions (Figure 1(a)), exhibiting inhibitory effect at lower doses, similar to that of papaverine (Figures 1(c) and 1(d)), whereas verapamil showed higher potency against high K^+^ when compared with carbachol (Figure 1(d)). Similarly, Flaxseed oil exhibited inhibitory effect against CCh- (1 *μ*M) induced contractions at a higher concentration of 5 mg/ml (Figure 1(b)); hence, Fs.Cr was more potent against CCh- (1 *μ*M) induced contractions (*p* < 0.01) as compared to Flaxseed oil. In contrast, Flaxseed oil was found selective at the activation of K^+^ channels, which was absent in Fs.Cr at the tested doses, whereas its effect against high K^+^ was negligible as previously reported [6] (Figure 1(b)).

Any material that will inhibit CCh-induced contraction is either PDEI, anticholinergic, or both [26]. Hence, Fs.Cr was further explored for its anticholinergic and PDEI mechanisms.

#### 3.3.1. PDEI Activity of Fs.Cr and Flaxseed Oil

**Figure 2:**
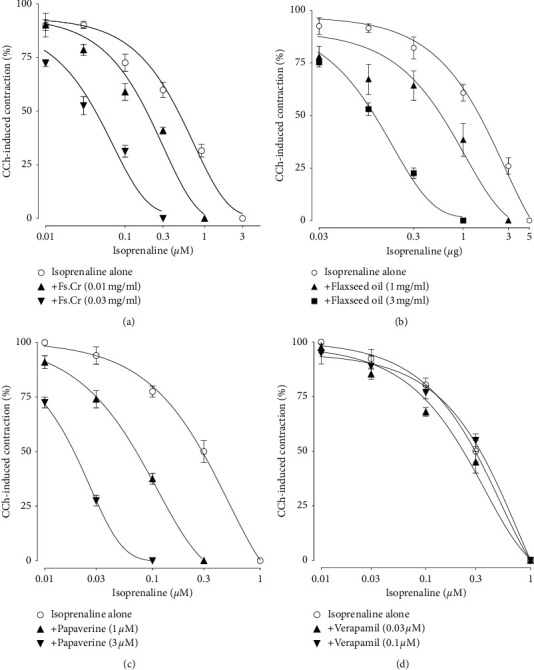
Concentration-response curves (CRCs) of isoprenaline.

To test for the presence or absence of PDEI-like effect, inhibitory CRCs of isoprenaline were constructed against CCh-induced sustained contractions (Figures 2(a)–2(d)) in the presence and absence of Fs.Cr (Figure 2(a)) and Flaxseed oil (Figure 2(b)) in rabbit jejunal tissues. Pretreatment of the jejunal tissues with increasing concentrations of Fs.Cr (0.01 and 0.03 mg/ml) and Flaxseed oil (1 mg/ml and 3 mg/ml) shifted the isoprenaline-induced inhibitory CRCs against CCh (1 *μ*M) to the left, indicating PDEI, similar to papaverine at 1 and 3 *μ*M (Figure 2(c)), whereas there was no shift in the case of verapamil (0.03 and 0.1 *μ*M) (Figure 2(d)). Due to the strong PDEI effect of Fs.Cr, it was further explored for in-depth mechanisms for its subtype and the associated increase in cAMP levels.

**Figure 3:**
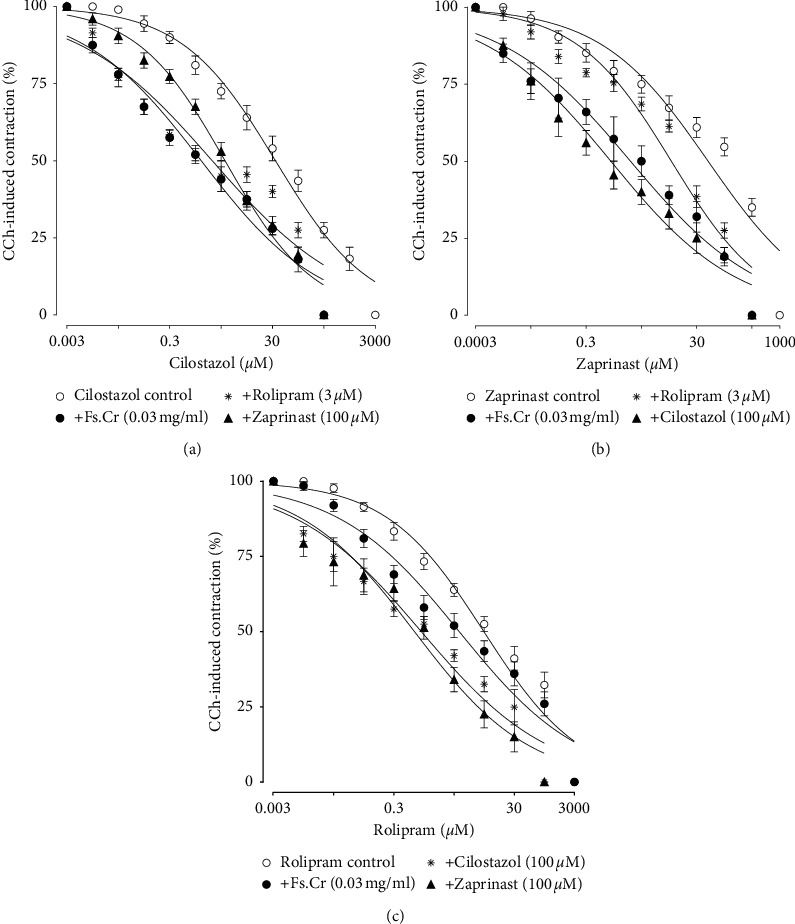
((a)–(c)) Effect of Fs.Cr on the relaxant responses of cilostazol (a), zaprinast (b), and rolipram (c) in rabbit jejunum precontracted with CCh (1 *μ*M).

Since PDE inhibitory effect was the predominant mechanism for the antispasmodic activity of Fs.Cr, it was further studied for the possible subtype of PDE involved. PDEI are expected to inhibit CCh-induced sustained contractions. Hence, at first, the effect of different PDE inhibitors' subtypes against CCh-induced contraction, with and without pretreatment with Fs.Cr, was recorded (Figures 3(a)–3(c)).

Cilostazol, a selective inhibitor of PDE-3, showed a concentration-dependent inhibitory response against CCh-induced contraction as evident in Figures 3(a). Pretreatment with Fs.Cr potentiated the inhibitory effect of cilostazol (*p* < 0.001), as evident by a leftward shift in the curve along with a significant reduction in EC_50_ values (additive effect) (Table 4). A similar pattern was evident when effect of cilostazol was measured with preincubation of rolipram (PDE-4 subtype inhibitor) and zaprinast (PDE-5 subtype inhibitor) indicating that Fs.Cr may have inhibited a PDE subtype other than cilostazol. A similar leftward shift was evident when inhibitory CRC was constructed against zaprinast, in presence of Fs.Cr and other standard PDE inhibitors.

Rolipram, a selective inhibitor of PDE-4, inhibited the contraction induced by CCh in a concentration-dependent manner. Interestingly, the inhibitory response of rolipram was not potentiated by pretreatment with Fs.Cr, whereas it was significantly potentiated by pretreatment with cilostazol (*p* < 0.001) and also zaprinast (*p* < 0.001) as evident in Figures 3(b). Hence, it is concluded that Fs.Cr might be inhibiting PDE-4 subtype similar to rolipram, a standard PDE-4 inhibitor.

**Figure 4:**
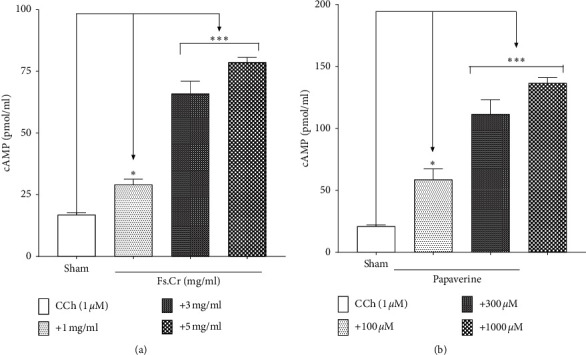
Effect of Fs.Cr (a) and papaverine (b) on the cyclic nucleotide content of rabbit jejunum.

Since cAMP levels are increased when PDE enzyme is inhibited in the tissues [30], we also estimated the cAMP levels in the control CCh-treated tissues and compared them with Fs.Cr- and papaverine-pretreated jejunal tissues. Untreated tissues showed 16.89 ± 1.42 pmol of cAMP/ml of tissue homogenate. Compared to this, Fs.Cr after treatment with 1, 3, and 5 mg/ml of Fs.Cr against CCh-induced contraction showed cAMP levels up to 29.11 ± 1.62 (*p* < 0.05), 65.99 ± 1 (*p* < 0.001), and 78.73 ± 1.17 (*p* < 0.001) pmol/ml, respectively, as evident in Figure 4(a). The papaverine control showed similar pattern of the rise in the cAMP levels with control CCh-treated tissues showing cAMP levels of 21.01 ± 1.75 pmol/ml, whereas treatment with 1 *μ*M and 3 *μ*M of papaverine in CCh-contracted tissues increased the cAMP levels to 58.7 ± 4.2 pmol/ml (*p* < 0.05) and 91.89 ± 3.4 (*p* < 0.001) pmol/ml, respectively, as evident in Figure 4(b).

#### 3.3.2. Anticholinergic Activity of Fs.Cr and Flaxseed Oil

**Figure 5:**
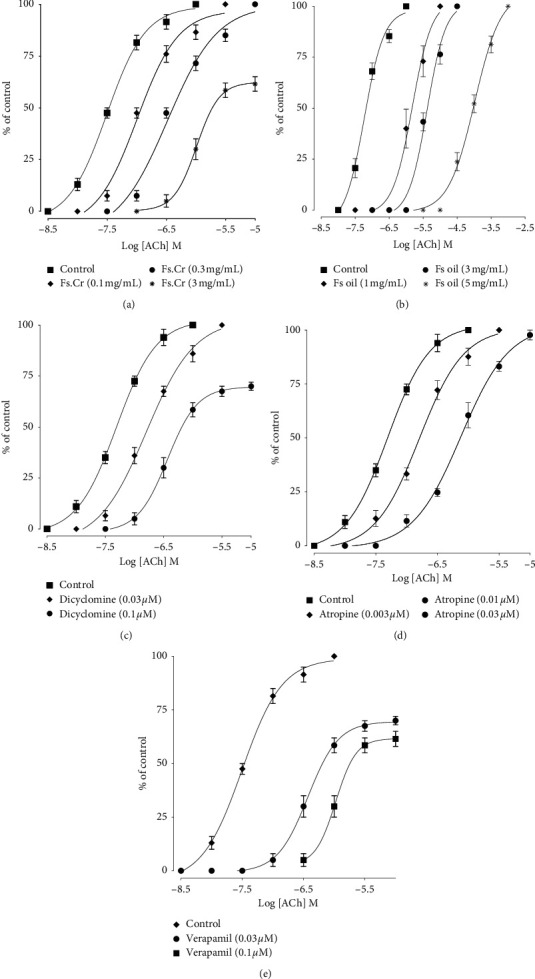
Concentration-response curves of Ach in the absence and presence of the increasing concentrations of (a) Fs.Cr, FS oil (b), dicyclomine (c), atropine (d), and verapamil (e) in isolated rabbit jejunum preparations.

Besides PDEI, CCh-induced contraction is also inhibited by anticholinergics; therefore, Flaxseed extract and oil were further tested in rabbit jejunal tissues to see if anticholinergic activity exists. Pretreatment of Fs.Cr (0.1 and 0.3 mg/ml) shifted Ach-induced bolus CRCs to the right in parallel manner without suppression of the maximal response, in a concentration-dependent manner as shown in Figure 5(a). With further increase in the dose of extract at 1 mg/ml, the rightward shift in Ach CRCs was nonparallel with suppression in maximal response. Dicyclomine, which is known to exhibit a cholinergic antagonist effect at a lower dose and CCB at a higher dose, was used as a standard and showed a similar rightward parallel shift of Ach CRCs at 0.03 *μ*M (*p* < 0.05) and a rightward nonparallel shift due to suppression of the maximal response at 0.1 *μ*M, as shown in Figure 5(c).

Flaxseed oil shifted Ach-induced bolus CRCs to the right in parallel manner without suppression of the maximal response, at higher doses of 1, 3, and 5 mg/ml in a concentration-dependent manner as shown in Figure 5(b), similar to atropine, as shown in Figure 5(d). This further indicates that Fs.Cr was more potent anticholinergic than Flaxseed oil.

### 3.4. Antibacterial Activity

**Figure 6:**
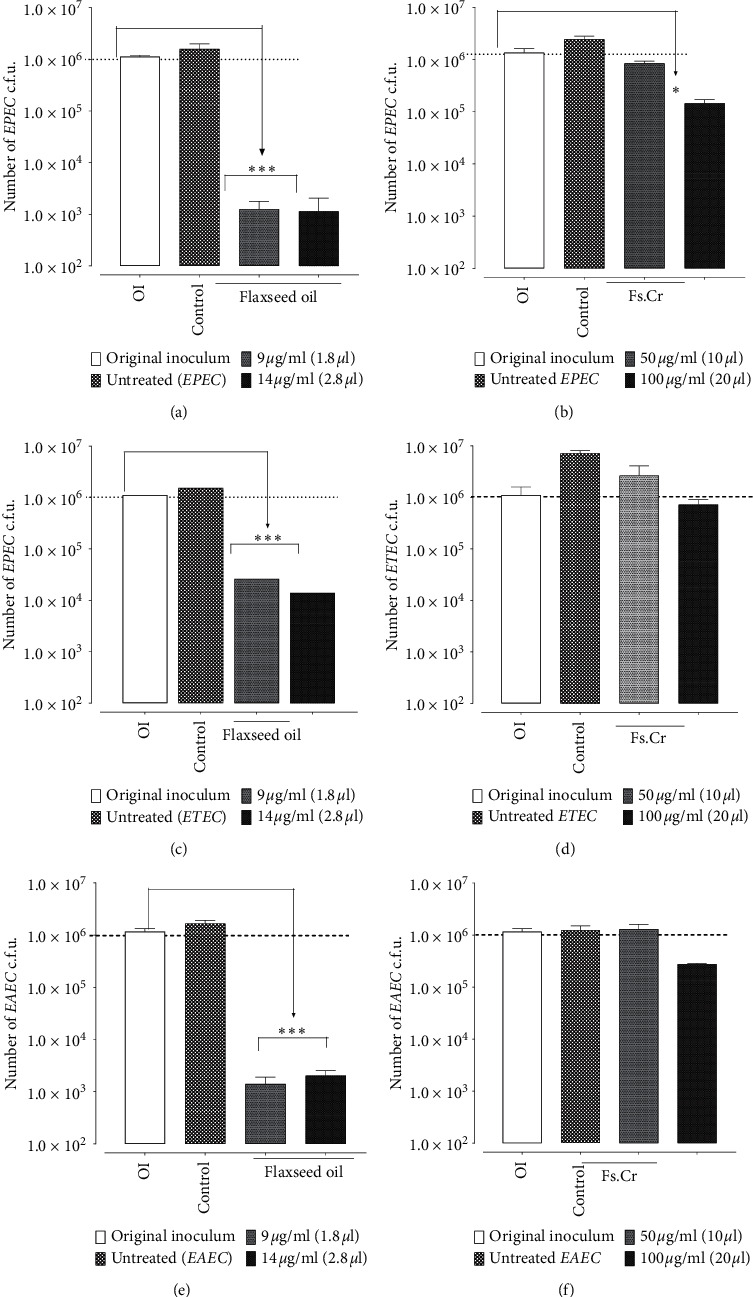
Bar charts show colony counts of ∼10^6^ EPEC (a, d); ETEC (b, e); and EAEC (c, f) incubated with and without Flaxseed oil (a–c) and Flaxseed extract (Fs.Cr) (d–f).

Both Flaxseed extract (Fs.Cr) and oil were tested *in vitro* for their effect against EPEC, ETEC, and EAEC. Flaxseed oil was more potent and efficacious against all the tested bacteria, as compared to Fs.Cr. The bactericidal effect of Flaxseed oil increased dose-dependently at 9 and 14 *μ*g/ml (Figures 6(a)–6(c)), whereas the effect of Fs.Cr was only evident against EPEC at 100 *μ*g/ml, and no effect was observed against ETEC and EAEC (Figures 6(d)–6(f)). The colony counts (c.f.u.) are elaborated in Supplementary Table 3. Hence, the minimal effective dose for antibacterial activity against these bacteria is almost 100 *μ*g/ml for Fs.Cr and 9 *μ*g/ml for Flaxseed oil.

Since Fs.Cr did not exhibit bactericidal activity against ETEC and EAEC, a possibility of bacteriostatic mechanism was tested by increasing the incubation time from 2 to 16 hrs (to provide time for bacteria to grow). Doses of 5 and 12.5 mg/ml of Fs.Cr were used for assay time of 16 hrs, whereas doses of 0.05 and 0.1 mg (50 and 100 *µ*g/ml) of Fs.Cr were used when assay time was 2 hrs. The analysis is shown in Supplementary Figures 2–4 (A-B).

## 4. Discussion

Inflammatory bowel disease (IBD) is a manifestation of multiple dysregulations acting simultaneously; therefore, it needs to be catered from different ends for an effective disease control. Multitargeted approach has gained a lot of interest in IBD therapeutics [1]; therefore, we explored the various pharmacological targets of Flaxseed in order to rationalize its medicinal use in IBD.

Our preliminary studies have shown that Flaxseed extract (Fs.Cr) was effective against 6% AA-induced colitis in mice due to its antioxidant and anti-inflammatory activities [4]. However, this does not represent the potential of whole Flaxseed, as the extract, so prepared, mainly constitutes polar compounds of Flaxseed [5]. Hence, we studied different mechanisms of both Flaxseed extract (known to contain polar constituents) and oil (known to contain nonpolar constituents) and then compared their effects on various parameters implicated in IBD.

The first step was to identify the effectiveness of Flaxseed oil against IBD and compare it with Flaxseed extract (Fs.Cr). It was evident with parameters such as mortality rate, disease activity index (DAI), and microscopic scoring that Flaxseed oil was as effective in ameliorating the severity of IBD in mice model as Fs.Cr; however, there were some differences. One of the differences was that Fs.Cr was more potent than Flaxseed oil in producing these effects. Flaxseed oil was more efficacious in reducing ulcer size as compared to Fs.Cr reduction in neutrophil, and lymphocytic infiltration was slightly better with Fs.Cr than Flaxseed oil. Fs.Cr was also more potent and efficacious in promoting mucin production than Flaxseed oil. Despite these advancements, the mortality rate of Flaxseed oil was less as compared to Fs.Cr Differences in inflammatory and mucosal protective parameters in the extract and oil indicate that both the polar and nonpolar fractions of Flaxseed have the potential to mediate mucosal protective effect in the IBD model of mice and may provide a synergistic effect when given as a whole seed. This seems true as Flaxseed and its oil showed clinical improvement in ulcerative colitis patients in one of the latest reported studies [31]. On the contrary, our current findings are in contrast to an earlier report, where 10% Flaxseed diet aggravated the severity of dextran sodium sulfate- (DSS-) induced colitis model of mice [32]. One of the possibilities for this could be that Flaxseed meal has a laxative effect [33], which is known to aggravate colitis. Nevertheless, improvement in clinical studies in UC patients shows that this may not be the case, or this aspect may vary depending on the severity of the disease. In the clinical study, patients who were not on corticosteroids and/or immune modulators were opted for, which means that the recruited UC patients have limited disease severity. More animal and clinical studies are needed to clarify this aspect.

The other part of our study aimed to identify the mechanisms of Flaxseed in terms of its antispasmodic effect. Interestingly, not only did we find the antispasmodic mechanisms, but we also got some lead for its anti-inflammatory effect. We found that both Fs.Cr and Flaxseed oil caused complete inhibition of CCh-induced contraction, compared with other spasmogens, which is indicative of PDE inhibitory activity and/or anticholinergic activity [26, 34]. Upon further experimentation, it was evident that Fs.Cr not only exhibited a stronger PDEI, but also increased the tissue cAMP levels. Hence, we further aimed to identify which subtype of PDEI receptor was inhibited. It was found that Fs.Cr mediated the similar inhibitory pathway for PDE enzyme as rolipram, a standard PDE4 inhibitor. This was identified because Fs.Cr and rolipram given simultaneously could not produce synergistic inhibitory effect against CCh-induced contraction, whereas other PDEI, when coadministered with Fs.Cr, potentiated the effect, indicating that perhaps rolipram and Fs.Cr worked through the same subtype of PDE enzyme. This interpretation is further supported by previous studies where the effect of one of the subtypes of PDE inhibitors has been potentiated against CCh-induced contraction when pretreated with the other subtypes of PDE inhibitor [23, 24, 30]. This finding has a very important implication because blocking PDE-4 enzyme has been proposed as one of the novel approaches to target IBD. PDE-4 enzymes are present on inflammatory cells, and their inhibition prevents mucosal damage and subsequent inflammatory cell infiltration by increasing cAMP [35]. Our findings are also relatable because subsequently we were able to show the increased level of cAMP in the Fs.Cr-treated tissues, which is the end result of PDE-4 subtype inhibition. Interestingly, in the HPLC fingerprints of Fs.Cr (unpublished), one of the peaks matched quercetin, a flavonoid that has lately been identified as a potential PDE-4 inhibitor [36], and has been shown to ameliorate the severity of acetic acid colitis in rats [37].

Fs.Cr was the most potent against CCh-induced contraction, resembling papaverine. It was however different in that it showed some selectivity against high K^+^, like dicyclomine, whereas papaverine showed similar inhibition against both spontaneous and high K^+^-induced contractions. This raised the possibility of an additional anticholinergic along with Ca^+2^ antagonist-like activity, similar to dicyclomine component at a higher dose. Interestingly, when tested for its anticholinergic activity, Fs.Cr, at 0.3 and 1 mg/ml concentrations, caused the rightward shift of the CRCs of Ach without suppression of the maximum response, a classic pattern of a competitive antagonist [27] such as dicyclomine. Expectedly, at the higher concentration of 3 mg/ml, Fs.Cr further shifted the CRCs of Ach to the right but suppressed the maximal response, possibly due to the Ca^+^ channel antagonist-like mechanism, which has also been reported earlier [7]. Hence, Fs.Cr has been proved to exhibit multiple antispasmodic mechanisms ranging from PDEI-like effect at a lower concentration, followed by anticholinergic at a relatively higher concentration and Ca^+^ antagonist-like effect at a further higher concentration. Contrary to the extract, Flaxseed oil mediated antispasmodic effect predominately through K^+^ channel opener (KCO) activity [6]. Both PDEI and anticholinergic activity were evident in oil at a relatively higher concentration, whereas CCB activity could not be observed. However, the possibility of some additional mechanisms cannot be ruled out.

With current findings, Flaxseed as a whole mediates antispasmodic action through multiple mechanisms including PDEI (subtype PDE-4), anticholinergic, KCO, and Ca^+2^ antagonist-like activities. All of these mechanisms are known to have a protective role in IBD [10, 11, 13] and thus provide an additional pharmacological basis for the effectiveness of Flaxseed's protective effect in IBD.

Besides therapeutic use of antispasmodics in IBD, antibacterials also are used as an adjunct because reducing the aggressive microbial load has shown improvement in clinical outcomes in patients with IBD [38, 39]. Therefore, in the current study, we targeted a few of the *E. coli* strains implicated in IBD. Flaxseed oil showed a bactericidal activity against all the three strains tested, but Fs.Cr was bactericidal against EPEC. This difference in efficacy and potency indicates that Flaxseed oil may have a different antibacterial mechanism or additional chemical constituent(s) with antibacterial activity.

The bactericidal effect of both Fs.Cr and Flaxseed oil against EPEC is a very important finding because EPEC's invasive level is more or less similar to AIEC strain (LF82) [13]. Moreover, EPEC has also been shown to sustain iodoacetamide-induced UC-like colitis in rats [40]. Hence, Flaxseed may reduce the invasive ability of *E.coli* in IBD patients and possibly its consequent manifestation that includes upregulation of NF-k*β* [41], modulation of IL-1*β*, IL-6, TNF-*α*, COX-2, and apoptosis, hence reducing inflammatory cascades. This effect coupled with the earlier reported antibacterial activity of Fs.Cr against *Salmonella typhi* [7], which is implicated in UC [39], and other Gram-positive and Gram-negative organisms [7, 42] provides another pharmacological basis for medicinal use of Flaxseed in IBD. This effectiveness of Fs.Cr could be correlated with the resemblance of HPLC peaks of Fs.Cr with two synthetic compounds that we have identified in one of our unpublished reports. Those compounds are ofloxacin and metronidazole, both of which have broad-spectrum coverage against aerobic and anaerobic pathogens [43]. However, the presence of some novel compounds is speculated based on 100% kill of VRE, which is resistant to conventional antibiotics. On the other hand, Flaxseed oil used in the current study exhibited a peak at later retention times (supplementary Figure 1), indicating the presence of polar constituents when analyzed through HPLC. This peak may correspond to alpha linolenic acid (ALA), as identified in previous reports [44], that has also exhibited effectiveness in the colitis model of mice [45]. Future studies will be directed toward the confirmation of these active constituents and their contribution to ameliorating effects in IBD.

This study is the first report on the effect of Flaxseed on bacteria implicated in IBD. Earlier, antibacterial effect of Flaxseed oil has been shown against *E.coli* [42], which is a nonpathogenic strain and hence cannot be compared. The current study shows that Flaxseed has bactericidal effect against pathogens implicated in IBD, which may contribute to managing immune dysregulation from a different end than cytokine modulation.

## 5. Conclusion

Based on the current findings, we understand that both Flaxseed oil and extract are effective against IBD model of mice, with possibly different mucosal protective mechanisms. Both the extract and the oil exhibit antispasmodic activity with partly similar mechanisms. Fs.Cr has predominant PDE inhibitor-like effect and weaker Ca^++^ antagonist effect, whereas Flaxseed oil has predominant KCO-like effect and weak PDEI effect. Both the extract and the oil are effective against microbes implicated in IBD, with a stronger antibacterial activity in Flaxseed oil. This, coupled with previously reported cytokine modulatory and antioxidant effects of Fs.Cr, indicates that the whole seed must encompass the potential of both the extract and the oil and therefore shall give an augmented response in IBD. Hence, Flaxseed may become a choice of remedy that helps to cater the basic pathophysiological tenets of IBD, which involve immune dysregulation and barrier defects as well as microbial dysregulation.

## Figures and Tables

**Table 1:** Disease activity index (DAI) scoring criteria.

Parameter	Scoring criteria
Weight loss	1–5%, 6–10%, 11–20%, > 21% = 0, 1, 2, 3
Stool	Normal, soft but still formed, very soft, diarrhoea = 0, 1, 2, 3
Bleed/occult	Normal, hemoccult positive, blood traces in stool visible, gross bleeding = 0, 1, 2, 3
DAI	(Weight loss + stool consistency + rectal bleeding)/3

**Table 2:** Comparison of mortality rate and DAI of acetic acid-induced colitis in mice.

	Mortality rate (%)		DAI (%)	
	Oil	Extract	Oil	Extract
Untreated	58.33 ± 4.23#	50 ± 5.01%	2.17 ± 0.06	2.11 ± 0.03#
150 mg/kg	55 ± 12%	33.33 ± 5%^*∗*^	2.1 ± 0.08	1.17 ± 0.01^*∗*^
300 mg/kg	33.33 ± 6%^*∗∗*^	25 ± 8%^*∗∗*^	1.80 ± 0.02^*∗∗*^	0.71 ± 0.21^*∗∗*^
500 mg/kg	8.33 ± 5%^*∗∗∗*^	33 ± 6%^*∗∗∗*^	0.93 ± 0.01^*∗∗∗*^	0.97 ± 0.23^*∗∗*^

Values given are means ± SEM of 3 experiments (*n* = 12). One-way ANOVA was used for statistical analysis followed by Tukey's posttest. ^*∗*^*p* < 0.05, ^*∗∗*^*p* < 0.01; ^*∗∗∗*^*p* < 0.001 compared with the untreated group (#); all the drugs were administered intraperitoneally for 7 days. Colitis was induced on the 6^th^ day with intrarectal administration of 0.1 ml 6% acetic acid. Mice were assessed 24 hrs after induction of colitis. DAI [18] = (weight loss + stool consistency + rectal bleeding)/3; mortality rate = (mice died in a group/total number of mice in that group) × 100. DAI: disease activity index.

**Table 3:** Comparison of histopathological scoring for Flaxseed oil and extract.

		Oil % reduction from control	Extract % reduction from control	Oil vs. extract
Vilaseca score	Ulceration			
	Untreated			
	150 mg/kg	3.13%	24%	^*∗∗∗*^
	300 mg/kg	25%	56%^*∗*^	^*∗∗∗*^
	500 mg/kg	77.5%^*∗∗*^	33.14%	^*∗∗∗*^
	Inflammation			
	150 mg/kg	2.27%	8.5%	^*∗∗∗*^
	300 mg/kg	13.64%	50%^*∗∗∗*^	^*∗∗∗*^
	500 mg/kg	35.46%^*∗∗*^	30%	NS
	Depth of lesion^1^			
	150 mg/kg	7.69%	23.07%^*∗*^	
	300 mg/kg	23.08%^*∗∗*^	55%^*∗∗∗*^	^*∗∗∗*^
	500 mg/kg	42.31%^*∗∗∗*^	38.47%^*∗∗∗*^	NS
Neurath score	150 mg/kg	8.82%	33.04%	^*∗∗*^
	300 mg/kg	26.47%^*∗∗*^	55.18%^*∗*^	^*∗∗∗*^
	500 mg/kg	55.89%^*∗∗∗*^	51.15%^*∗∗*^	NS
Mucin score (%)	150 mg/kg	2.85%	25%	^*∗∗∗*^
	300 mg/kg	35.71%^*∗*^	75%^*∗∗∗*^	^*∗∗∗*^
	500 mg/kg	42.28%^*∗∗*^	91.66%^*∗∗∗*^	^*∗∗∗*^

Neurath score [19]: score 0 = no leukocyte infiltration; score 1 = low level of leukocyte infiltration; score 2 = moderate level of leukocyte infiltration; score 3 = moderate level of leukocyte infiltration, high vascular density, and thickening of colon wall; score 4 = transmural leukocyte infiltration, loss of goblet cells, high vascular density, and thickening of the colon wall. Vilaseca Score [20]: total score = 9 ulceration: 1 [no ulcer, epithelialization; small ulcers < 3 mm; large ulcers ≥ 3 mm = score 0, 1, 2, respectively]; inflammation: 3 [none, mild, moderate, severe; 0, 1, 2, 3]; depth of lesion: 3 [none, mucosa, muscularis propria, serosa; 0, 1, 2, 3]; mucin score [21]: score 4 [<1% of stained cells; 1–30% of stained cells (low level of staining); 30–80% of stained cells (medium level of staining); up to 80% of stained cells = score 0, 1, 2, 3]. One-way ANOVA followed by Tukey's posttest was used for analysis. Values expressed are means ± SEM (*n* = 6). ^*∗*^*p* < 0.05, ^*∗∗*^ = *p* < 0.01; ^*∗∗∗*^ = *p* < 0.001 vs AA. #Diseased control group. Two-way ANOVA followed by Bonferroni's posttest was used for comparing the effect of corresponding doses of Flaxseed oil and extract. All the drugs were administered i.p. Colitis was induced by IR administration of 0.1 ml 6% acetic acid during anaesthesia. Tissues were embedded in formalin for another 24 hrs, then processed, and stained with H and E and PAS-AB staining. i.p.: intraperitoneally; IR: intrarectal; H and E: hematoxylin and eosin; PAS-AB: periodic acid-Schiff-Alcian blue.

**Table 4:** EC_50_ values of different PDE-subtype inhibitors with and without Fs.Cr pretreatment.

PDE inhibitor				
Cilostazol (*µ*M)	Control	+Fs.Cr	+Rolipram	+Zaprinast
EC_50_	156.3#	0.91^*∗∗∗*^	0.15^*∗∗∗*^	2.83^*∗∗∗*^
CI	76.32 to 319.9	0.35 to 2.35	0.06 to 0.39	1.48 to 5.41
Rolipram (*µ*M)	Control	+Fs.Cr	+Cilostazol (*µ*M)	+Zaprinast
EC50	12.41#	10.34	2.421^*∗∗∗*^	1.61^*∗∗∗*^
CI	6.42 to 23.98	5.49 to 19.46	1.21 to 4.83	0.96 to 2.70
Zaprinast (*µ*M)	Control	+Fs.Cr	+Rolipram	+Cilostazol
EC50	146.3#	3.59^*∗∗∗*^	19.28^*∗∗∗*^	0.38^*∗∗*^
CI	74.45 to 287.4	1.381 to 9.37	11.91 to 31.21	0.15 to 0.96

EC_50_: effective concentration to produce 50% response; CI: confidence interval; Fs.Cr: aqueous-methanolic crude extract of Flaxseed (70; 30). All concentrations are in *µ*M. ^*∗∗∗*^*p* < 0.001; ^*∗∗*^*p* < 0.01; one-way ANOVA was done followed by Tukey's posttest to compare the significance of means ± SEM of controls with other groups. ^#^All treatment groups are compared with Control.

## Data Availability

The data and materials supporting this study are available from the corresponding author upon request.

## Abbreviations

%: Percent
°C: Degree centigrade
*µ*g: Microgram
*μ*M: Micromolar
*μ*L: Microliter
AA: Acetic acid
Ach: Acetylcholine chloride
Ca+2: Calcium
cAMP: Cyclic adenosine monophosphate
CCh: Carbachol
CD: Crohn's disease
c.f.u.: Colony forming unit(s)
COX-2: Cyclooxygenase 2
CRCs: Concentration-response curves
DAI: Disease activity index
DMSO: Di methyl sulfoxide
EAEC: Enteroaggregative *Escherichia coli*
ECACU: Ethics Committee for Animal Care and Use
EPEC: Enteropathogenic *Escherichia coli*
ETEC: Enterotoxigenic *Escherichia coli*
Fs.Cr: Aqueous-methanolic crude extract of *Linum usitatissimum* seeds
Gm: Gram
GI: Gastrointestinal
H and E: Hematoxylin and eosin
K+: Potassium
KCO: Potassium channel opener
Hr(s): Hour(s)
IBD: Inflammatory bowel disease
IL-1*β*: Interleukin-1 beta
IL-6: Interleukin-6
i.p.: Intraperitoneally
IR: Intrarectal
Kg: Kilogram
LB: Luria-Bertani broth
MeOH: Methanolic
mL: Milliliter
mM: Millimolar
NF-k*β*: Nuclear factor kappa-beta
OD: Optical density
OI: Original inoculum
PAS-AB: Periodic acid-Schiff-Alcian blue
PDE-4: Phosphodiesterase-4 subtype
PDEI: Phosphodiesterase inhibitors
Sec: Second
SEM: Standard error of mean
TNF-*α*: Tumour necrosis factor-alpha
UC: Ulcerative colitis
v/v: Volume/volume
w/w: Weight by weight.

## Supplementary Materials

Supplementary Materials Supplementary Table 1: Multiple scoring criteria to assess microscopic damage in the colon of mice. Supplementary Table 2: Microscopic scoring of H and E stained and PAS-AB stained colon sections of mice after treatment with Fs.Cr and Flaxseed oil. Supplementary Table 3: Colony count of EPEC, ETEC. and EAEC after treatment with Fs.Cr and Flaxseed oil. Supplementary Figure 1: HPLC fingerprint analysis of Flaxseed oil. Supplementary Figure 2 (A-B): Bar charts show effect on colony counts of ∼106 enteropathogenic *E. coli* (EPEC) incubated with and without Fs.Cr for 16 hrs (A) and 2 hrs (B). Doses of 1 and 2.5 mg of Fs.Cr were used for assay time of 16 hrs, whereas doses of 0.05 and 0.1 mg (50 and 100 *µ*g/ml) of Fs.Cr were used when assay time was 2 hrs (B). Supplementary Figure 3 (A-B): Bar charts show effect on colony counts of ∼106 enterotoxigenic *E. coli* (ETEC) incubated with and without Fs.Cr for 16 hrs (A) and 2 hrs (B). Doses of 1 and 2.5 mg of Fs.Cr were used for assay time of 16 hrs, whereas doses of 0.05 and 0.1 mg (50 and 100 *µ*g/ml) of Fs.Cr were used when assay time was 2 hrs (B). Supplementary Figure 4 (A-B): Bar charts show the effect on colony counts of ∼106 enteroaggregative *E. coli* (EAEC) incubated with and without Flaxseed extract (Fs.Cr) for 16 hrs (A) and 2 hrs (B). Doses of 1 and 2.5 mg of Fs.Cr were used for assay time of 16 hrs, whereas doses of 0.05 and 0.1 mg (50 and 100 *µ*g/ml) of Fs.Cr were used when assay time was 2 hrs (B).
