# Supplementary material for: Multiple Mechanisms of Flaxseed: Effectiveness in Inflammatory Bowel Disease
Source: Evid Based Complement Alternat Med. 2020 Jul 12;2020:7974835. doi: 10.1155/2020/7974835 (PMC7374215; doi:10.1155/2020/7974835)
Supplement: Supplementary Materials — Supplementary Table 1: Multiple scoring criteria to assess microscopic damage in the colon of mice. Supplementary Table 2: Microscopic scoring of H and E stained and PAS-AB stained colon sections of mice after treatment with Fs.Cr and Flaxseed oil. Supplementary Table 3: Colony count of EPEC, ETEC. and EAEC after treatment with Fs.Cr and Flaxseed oil. Supplementary Figure 1: HPLC fingerprint analysis of Flaxseed oil. Supplementary Figure 2 (A-B): Bar charts show effect on colony counts of ∼106 enteropathogenic E. coli (EPEC) incubated with and without Fs.Cr for 16 hrs (A) and 2 hrs (B). Doses of 1 and 2.5 mg of Fs.Cr were used for assay time of 16 hrs, whereas doses of 0.05 and 0.1 mg (50 and 100 µg/ml) of Fs.Cr were used when assay time was 2 hrs (B). Supplementary Figure 3 (A-B): Bar charts show effect on colony counts of ∼106 enterotoxigenic E. coli (ETEC) incubated with and without Fs.Cr for 16 hrs (A) and 2 hrs (B). Doses of 1 and 2.5 mg of Fs.Cr were used for assay time of 16 hrs, whereas doses of 0.05 and 0.1 mg (50 and 100 µg/ml) of Fs.Cr were used when assay time was 2 hrs (B). Supplementary Figure 4 (A-B): Bar charts show the effect on colony counts of ∼106 enteroaggregative E. coli (EAEC) incubated with and without Flaxseed extract (Fs.Cr) for 16 hrs (A) and 2 hrs (B). Doses of 1 and 2.5 mg of Fs.Cr were used for assay time of 16 hrs, whereas doses of 0.05 and 0.1 mg (50 and 100 µg/ml) of Fs.Cr were used when assay time was 2 hrs (B). [file 7974835.f1.pdf]

`

Supplementary TABLE 1: Multiple scoring criteria to assess microscopic damage in the colon of mice.:

Scoring type		Scoring variables	Microscopic Score
Neurath Score [1]		no leukocyte infiltration	0
		low level of leukocyte infiltration	1
		moderate level of leukocyte infiltration	2
		moderate level of leukocyte infiltration, high vascular density and thickening of colon wall	3
		transmural leukocyte infiltration, loss of goblet cells, high vascular \density and thickening of the colon wall.	4
Vilaseca Modified Score[2]	Ulceration	No ulcer, epithelization; Small ulcers,< 3 mm; Large ulcers > or equal to 3 mm = score 0,1,2, respectively	0
			1
			2
	Inflammation	None	0
		Mild	1
		Moderate	2
		Severe	3
	Depth of the lesion	None	0
		Submucosa	1
		Muscularis propria	2
		Serosa	3
Mucin scoring[4]		<1% of stained cells	0
		1-30% of stained cells; low level of staining;	1
		30-80% of stained cells; medium level of staining	2
		Up to 80% of stained cells; high level of staining	3

The semi-quantitative scoring system assessed the microscopic damage in AA induced colitic mice, treated with and without Fs.Cr, and then stained with H and E. This scoring was performed by a histopathologist who was blinded to the treatment. The criteria includes Neurath score (0-4)1(15), Vilaseca score (the sum of ulceration with maximum score 2, inflammation with maximum score 3 and depth of lesion with maximum score 3) (16). Goblet cells were assessed on the basis of level of mucin staining 4(17).

`

Supplementary Table 2: Microscopic Scoring score of H & E stained and PAS-Ab stained colon sections of mice after treatment with Fs.Cr and Flaxseed oil

		Flaxseed Oil Score (%)	Extract
**Vilaseca Score**	Ulceration		
	Untreated	1.6±0.1#	1.75±0.14#
	150 mg/kg	1.55±0.1 (96.87%)	1.33±0.19 (76%)
	300 mg/kg	1.2±0.16 (75%)	0.8±0.09** (44%)
	500 mg/kg	0.36±0.5** (22.5%)	1.11±0.2 *(63.42%)
	Inflammation		
	Untreated	2.2±0.05#	2±0.01#
	150 mg/kg	2.15±0.09 (97.72%)	1.83±0.18 (91.5%)
	300 mg/kg	1.9±0.2 (86.36%)	1±0.09*** (50%)
	500 mg/kg	1.42±0.22** (64.54%)	1.42±0.28 (57.5%)
	Depth of lesion		
	Untreated	2.29±0.05#	2.60±0.07#
	150 mg/kg	2.4±0.05 (92.3%)	2.1±0.06 * (76.92%)
	300 mg/kg	2±0.22** (76.92%)	1.2±0.05*** (45%)
	500 mg/kg	1.5±0.1*** (57.69%)	1.6±0.09*** (61.53%)
Neurath Score	Untreated	3.4±0.5#	3.48±0.65#
	150 mg/kg	3.1±0.09 (91.17%)	2.33±0.57 (66.95%)
	300 mg/kg	2.5±0.16** (73.52%)	1.56±0.5** (44.82%)
	500 mg/kg	1.5±0.02*** (44.11%)	1.70±0.03** (48.85%)
		Score (% increase)	Score (% increase)
**Mucin Score**	Untreated	1.4±0.2	1.2±0.37#
	150 mg/kg	1.36±0.04 (97.14%)	1.5±0.25 (125%)
	300 mg/kg	1.9±0.05*(135.71%)	2.1±0.25*** (175%)
	500 mg/kg	2±0.16** (142.28%)	2.3±0.5*** (191.66%)

ULCERATION: 1[No ulcer, epithelialization; Small ulcers, < 3 mm; Large ulcers > or equal to 3 mm = score 0, 1, 2, respectively]; INFLAMMATION: 2 [None, Mild, Moderate, Severe; 0, 1, 2, 3]; DEPTH OF LESION: 3 [None, Mucosa, Muscularis Propria, Serosa; 0, 1, 2, 3] NEURATH SCORE: 4 [No leukocyte infiltration; Low level of leukocyte infiltration; Moderate level of leukocyte infiltration; Moderate level of leukocyte infiltration, high vascular density and thickening of colon wall; Transmural leukocyte infiltration, loss of goblet cells, high vascular density and thickening of the colon wall = score 1; 2; 3; 4]; MUCIN SCORE: 4 [<1% of stained cells; 1-30% of stained cells (low level of staining); 30-80% of stained cells (medium level of staining); Up to 80% of stained cells (high level of staining) = score 0, 1, 2, 3]

`

Supplementary Table 3: Colony count of *EPEC, ETEC and EAEC* after treatment with Fs.Cr and Flaxseed oil

***EPEC* (c.f.u.)**			
OI	1.13E+06#	OI	1.36E+06#
Negative control	1.60E+06	Negative control	2.45E+06
Flaxseed oil (9 µg/ml)	1.27E+03***	Fs.Cr 50 µg/ml	8.50E+05
Flaxseed oil (14 µg/ml)	1.14E+03***	Fs.Cr 100 µg/ml	1.45E+05*
***ETEC* (c.f.u.)**			
OI	1.11E+06#	OI	1.11E+06
Negative control	1.53E+06	Negative control	7.23E+06
Flaxseed oil (9 µg/ml)	2.63E+04***	Fs.Cr 50 µg/ml	2.67E+06
Flaxseed oil (14 µg/ml)	1.40E+04***	Fs.Cr 100 µg/ml	7.25E+05
***EAEC* (c.f.u.)**			
OI	1.17E+06#	OI	1.17E+06
Negative control	1.67E+06	Negative control	1.25E+06
Flaxseed oil (9 µg/ml)	1.42E+03	Fs.Cr 50 µg/ml	1.30E+06
Flaxseed oil (14 µg/ml)	2.03E+03	Fs.Cr 100 µg/ml	2.77E+05

Assay used LB as an assay buffer with 2 hrs of incubation time. Bacteria corresponding to o.d. = 0.22 (~106 c.f.u.) were used for the assay. Values given are means±SEM of 3 experiments (n = 12). One way ANOVA was used for statistical analysis followed by Tukey's post test. **p<0.01; ***p<0.001 compared with untreated group (#). ; Fs.Cr = methanolic-aqueous crude extract of Flaxseed; OI = original inoculum

`

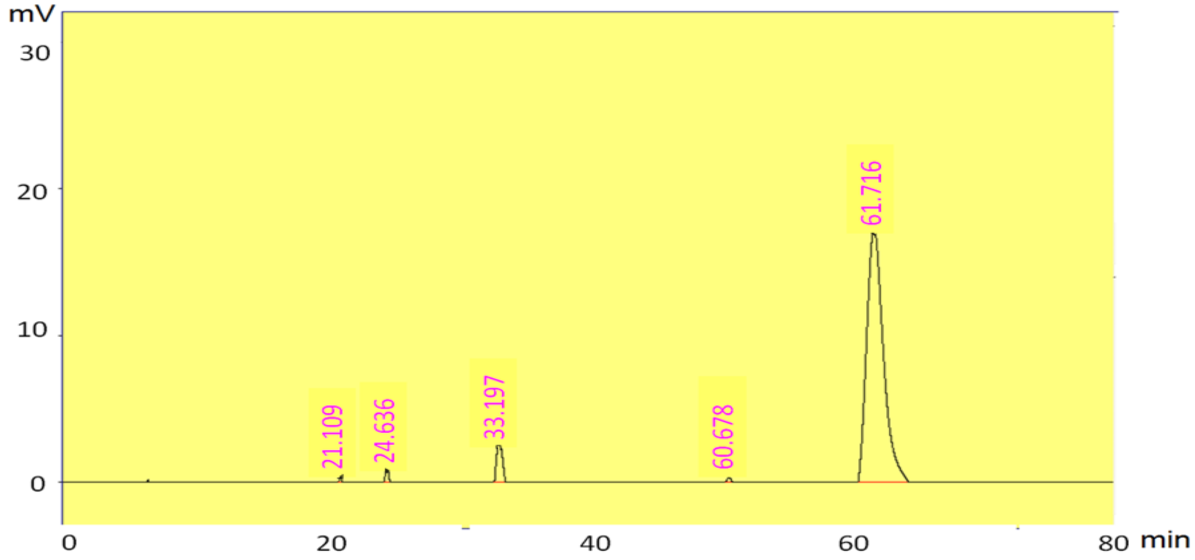

Supplementary Figure 1: Fingerprint analysis of Flaxseed oil.

`

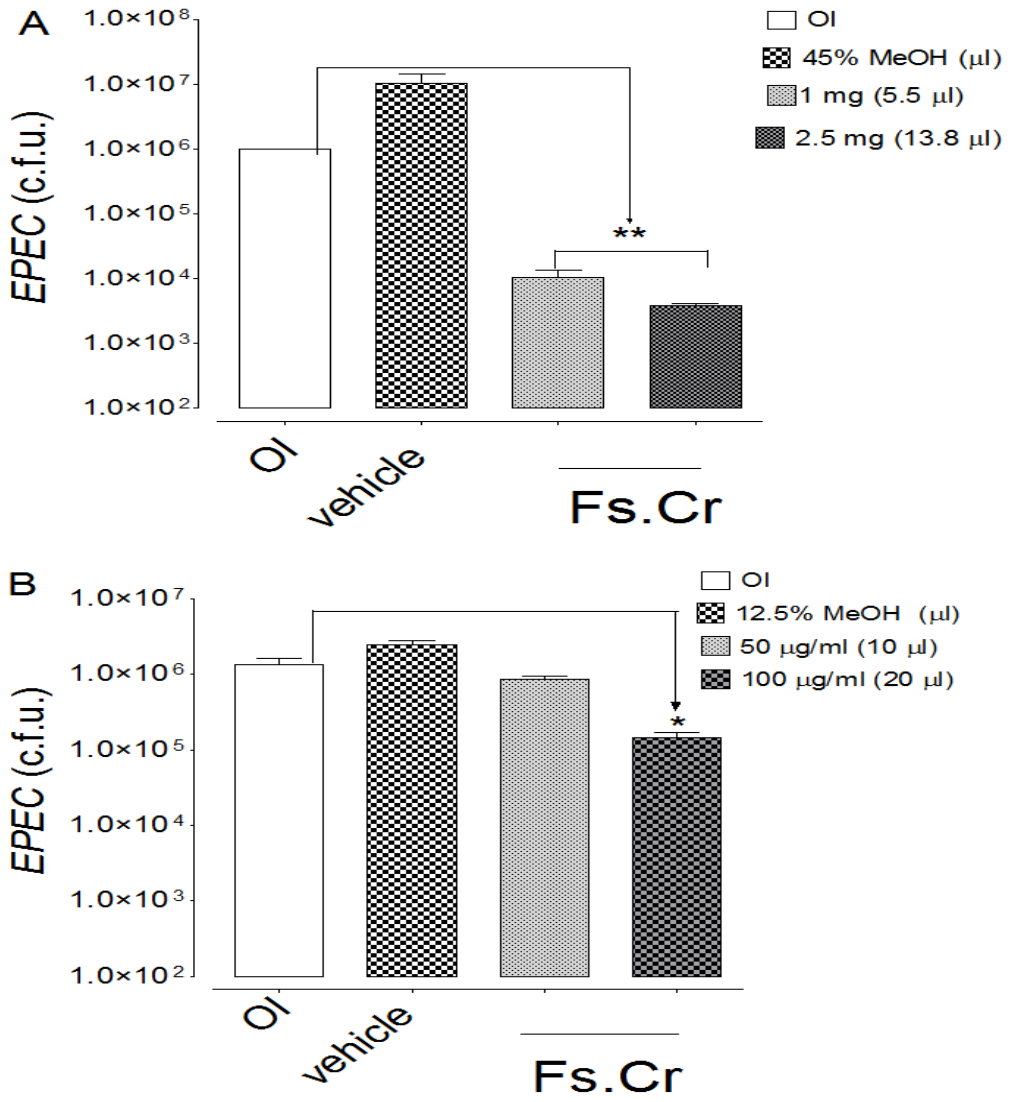

Supplementary Figure 2 (A-B); Bar-charts show effect on colony counts of ~10^6 enteropathogenic E. coli (EPEC) incubated with and without Fs.Cr for 16 hrs (A), and 2 hrs (B). Doses of 1 and 2.5 mg of Fs.Cr were used for assay time of 16 hrs, whereas, doses of 0.05 and 0.1 mg (50 and 100 µg/ml) of Fs.Cr were used when assay time was 2 hrs (B). The same corresponding volume of vehicle solvent was used in vehicle control group in 200 µl assay volume with Lurea broth (LB) as assay buffer, incubated at 37°C. Original inoculum (OI) is the approximate concentration of microbes plated before running assay (od = 0.22~106 c.f.u.). 100 µg/ml gentamycin was used as positive control not shown in graph (100% kill). The data are presented as the means±SEM of three independent experiments performed in duplicate. p-values were calculated by comparing OI and different extract concentrations using un-paired t- test. *; **; *** indicate $p<0.05$, $p<0.01$ and $p<0.001$ vs. OI.

[Symbols: Fs.Cr = Flaxseed extract; OI = original inoculum; od = optical density; LB = Luria broth; EPEC = enteropathogenic Eschericia coli; vehicle = 45% methanol (A); 12.5% MeOH for B].

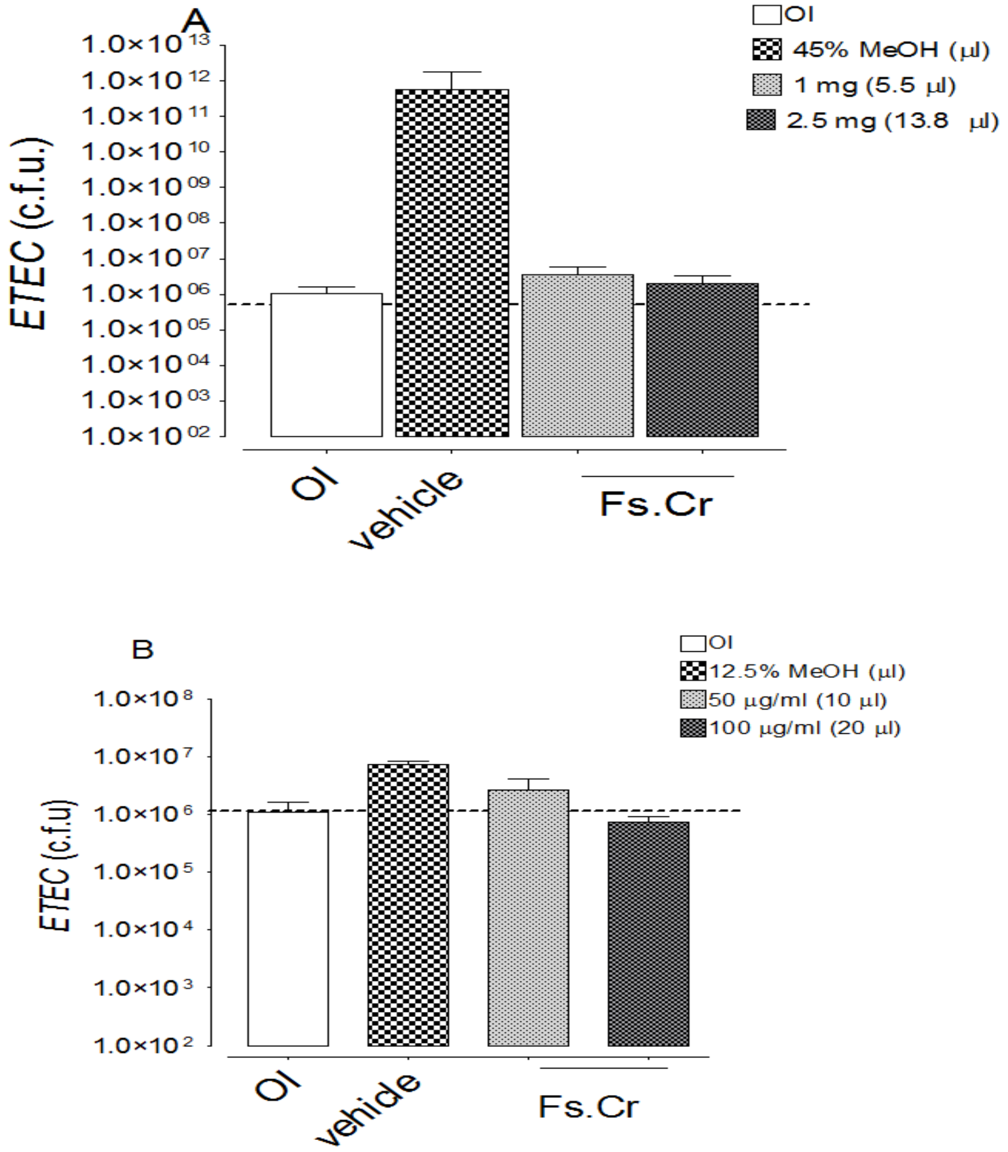

Supplementary Figure. 3 (A-B); Bar-charts show effect on colony counts of ~106 enterotoxigenic *E. coli* (*ETEC*) incubated with and without Fs.Cr for 16 hrs (A), and 2 hrs (B). Doses of 1 and 2.5 mg of Fs.Cr were used for assay time of 16 hrs, whereas, doses of 0.05 and 0.1 mg (50 and 100 µg/ml) of Fs.Cr were used when assay time was 2 hrs (B). The same corresponding volume of vehicle solvent was used in vehicle control group in 200 µl assay volume with Lurea broth (LB) as assay buffer, incubated at 37°C. Original inoculum (OI) is the approximate concentration of microbes plated before running assay (od = 0.22~10^6 c.f.u.). 100 µg/ml gentamycin was used as positive control not shown in graph (100% kill). The data are presented as the means±SEM of three independent experiments performed in duplicate. *p*-values were calculated by comparing OI and different extract concentrations using un-paired *t*- test.

[Symbols: Fs.Cr = Flaxseed extract; OI = original inoculum; od = optical density; LB = Luria broth; *ETEC* = enterotoxigenic *Eschericia coli*; vehicle = 45% methanol (A); 12.5% MeOH (B)].

`

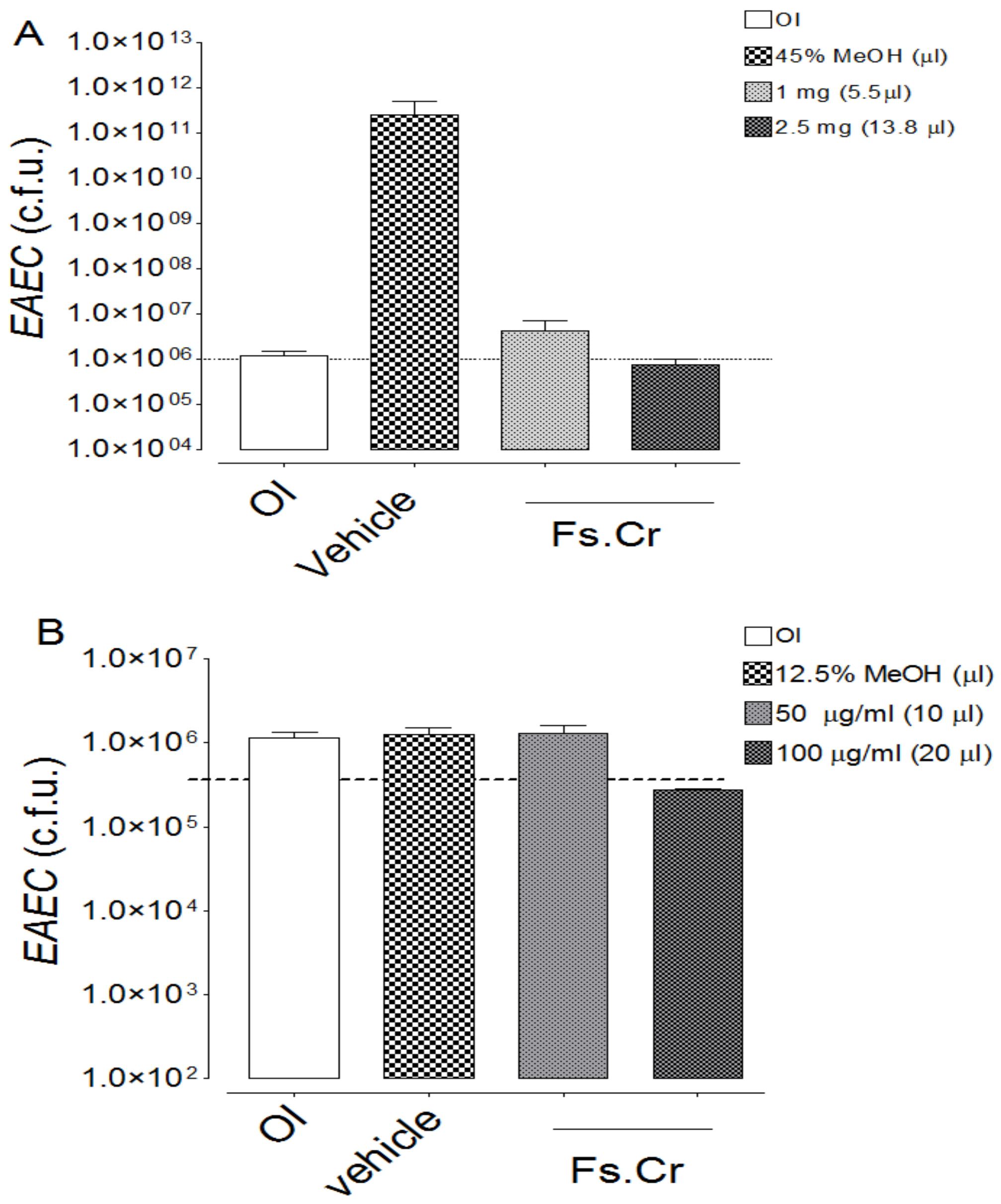

Supplementary Figure. 4 (A-B); Bar-charts show effect on colony counts of ~10^6 enteroaggregative *E. coli* (*EAEC*) incubated with and without Flaxseed extract (Fs.Cr) for 16 hrs (A) and 2 hrs (B). Doses of 1 and 2.5 mg of Fs.Cr were used for assay time of 16 hrs, whereas, doses of 0.05 and 0.1 mg (50 and 100 µg/ml) of Fs.Cr were used when assay time was 2 hrs (B). The same corresponding volume of vehicle solvent was used in vehicle control group in 200 µl assay volume with Lurea broth (LB) as assay buffer, incubated at 37°C. Original inoculum (OI) is the approximate concentration of microbes plated before running assay (od = 0.22~10^6 c.f.u.). 100 µg/ml gentamycin was used as positive control not shown in graph (100% kill). The data are presented as the means±SEM of three independent experiments performed in duplicate. *p*-values were calculated by comparing OI and different extract concentrations using un-paired *t*- test.

[Symbols: Fs.Cr = Flaxseed extract; OI = original inoculum; od = optical density; LB = Luria broth; *EAEC* = enteroaggregative *Eschericia coli*; vehicle = 45% methanol (A); 12.5% MeOH (B)].
